# Artificial intelligence, Internet addiction, and palliative care

**DOI:** 10.1192/j.eurpsy.2024.700

**Published:** 2024-08-27

**Authors:** S. Tei, J. Fujino

**Affiliations:** ^1^Department of Psychiatry, Kyoto university, Kyoto; ^2^Department of Psychiatry and Behavioral Sciences, Tokyo Medical and Dental University, Tokyo, Japan

## Abstract

**Introduction:**

Recent advances in artificial intelligence (AI) have recaptured and revised the essential roles of death in life and mind. However, their prospects and risks require further study. Because of the development of digital technologies (for example, AI-based chatbots), the process of bereavement may have become complex, immersive, and even addictive. Furthermore, AI-enabled generation of medical notes can ease the administrative burden for healthcare professionals; however, the clinical application of generative AI remains largely speculative.

**Objectives:**

This study aimed to illuminate the emerging concept and experience of death, bereavement, and addiction associated with cybernetics, thereby expanding their cognitive and ethical aspects.

**Methods:**

In this preliminary review, we performed a literature search to identify the current state-of-the-art literature on AI and Internet addiction. We also inspected the possible adaptations to pursue mental well-being with the modified death concept. We mainly searched the PubMed and Web of Science databases using relevant keywords. All retrieved studies were assessed for eligibility to reduce the selection bias.

**Results:**

Current cybernetics have meaningfully recontextualized death that allows interaction with deceased individuals (for example, scholars and artists) to establish their virtual, besides biological, existence using AI-based chatbots. Furthermore, AI consistently provides evidence-based answers to public health inquiries; nevertheless, it may offer unsuitable advice rather than referrals that can sometimes facilitate suicide or harm (instead of help) people in grief, thus requiring more fine-tuned governance. Accordingly, the maladaptive use of existing AI-related communication (such as metaverse characters) can increase Internet addiction prevalence and further complicate autonomy and self-motivation. In addition, excessive internet access is frequently associated with reduced self-control, cognitive flexibility, and exaggerated automatic processing.

**Conclusions:**

We are challenged to acknowledge the tradeoffs of AI and consider ways to compromise by employing flexible perspectives. The emerging concept of death affects or improves the conventional one. The potential advantages and pitfalls of AI-related technology must be carefully weighed against the profound effects they may have on people’s identities, relationships, and mental health. These issues require continued monitoring and assessment in light of the AI/cybernetic-related studies. We hope these results will inspire further research into the appropriate use of AI and palliative care, including suicide prevention, euthanasia, and grief management.

**Disclosure of Interest:**

None Declared

